# Supplementary material for: Bending strain engineering in quantum spin hall system for controlling spin currents
Source: Nat Commun. 2017 Jun 16;8:15850. doi: 10.1038/ncomms15850 (PMC5481753; doi:10.1038/ncomms15850)
Supplement: Supplementary Information — Supplementary Figures. [file ncomms15850-s1.pdf]

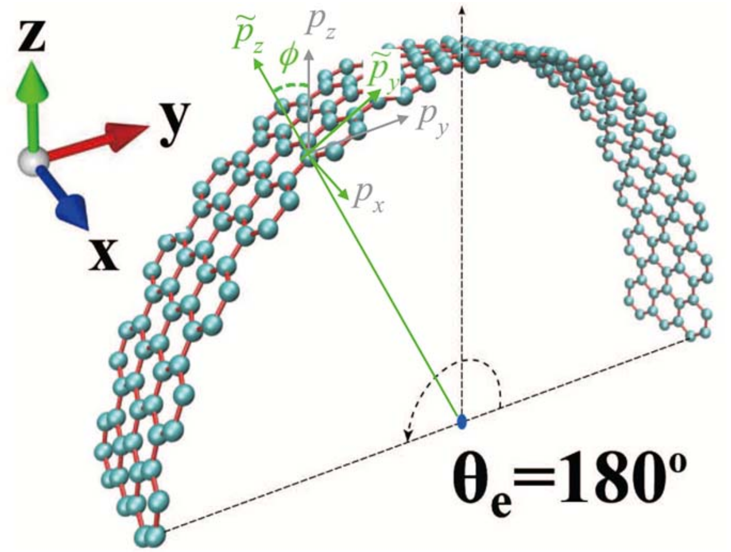

Supplementary Figure 1|The defination of rotation angle ϕ. After bending along x-axis, the directions of p_y, and p_z orbitals on each atomic site are rotated by an angle of ϕ from the gray arrows to the green ones (there is no rotation for p_x orbitals). ϕ is defined as the rotation angle between p_z (or p_y) orbitals before and after bending, i.e., the angle between gray and green arrows.

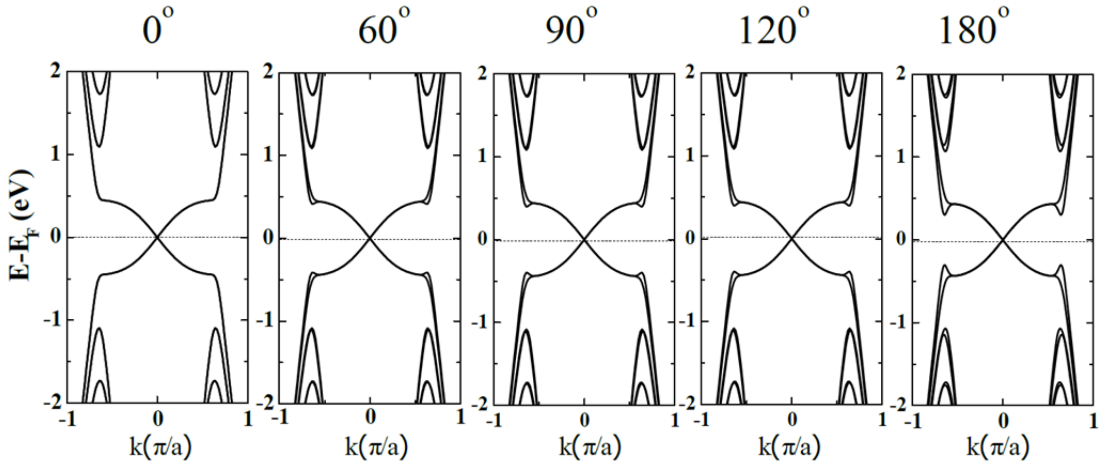

Supplementary Figure 2| The calculated band structures of zigzag-edge QSH nanoribbons (40 atoms/unitcell) as a function of bending angle θ_e. Generally, the value of charge conductance at a given energy is related to the number of bands crossing that energy. When the system is bent, the degeneracy of bulk structures is lifted, which results in additional band crossing at the energies around -0.5 eV and 0.5 eV, which make the number of band crossing the energy double. This additional band crossing can give rise to the increased charge conductance around -0.5 or 0.5 eV, as shown in Supplementary Figure 3.

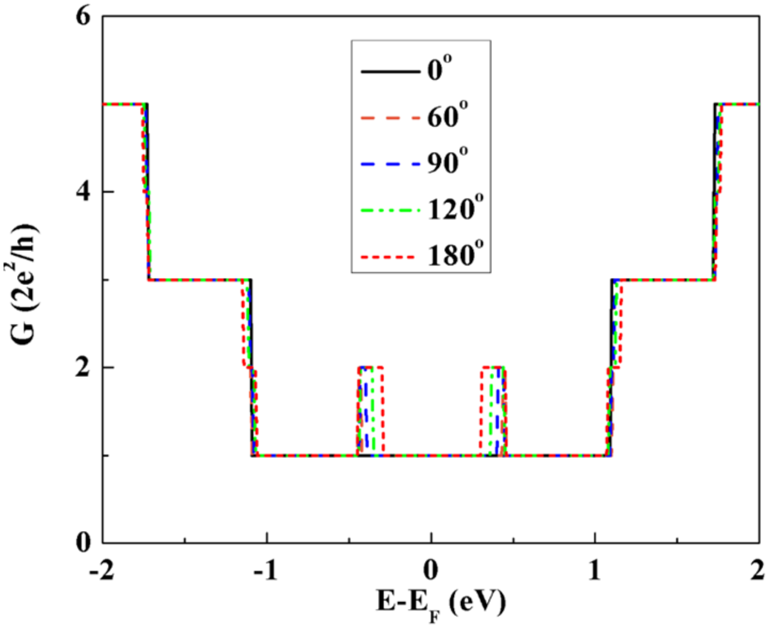

Supplementary Figure 3| The calculated charge conductance *G* for the zigzag-edge QSH nanoribbons a a function of bending angle θ_e. The calculated charge transmission coefficients *G* at different bending curvature demonstrate that the charge conductance *G* of a QSH nanoribbon is always quantized independent of θ_e, which is different from the spin conductance of a QSH nanoribbon.

Supplementary Note 1| Discussion for the details in Equation (4) and Equation (5) in the paper. In the rotated framework, $\tilde{H} = R^{\dagger} H_C R$, the total rotated spin z-component $\tilde{S}_z = R^{\dagger} S_z R$ is conserved. For every spin up/down for $\tilde{S}_z$, it is an ideal integer quantum Hall system and we can use TKNN formula to calculate the edge spin conductance $g_{L,R}^{\tilde{S}_z} = \frac{\tilde{C}_s}{2} \cdot \frac{e}{2\pi}$. Before the rotation of the framework, i.e. in the experimental framework, $S_y = sin(\phi)\tilde{S}_z$, $S_z = cos(\phi)\tilde{S}_z$, then we have $g^{Sy} = sin(\phi)\frac{\tilde{C}_s}{2} \cdot \frac{e}{2\pi}$, $g^{Sz} = \cos(\phi)\frac{\tilde{C}_s}{2} \cdot \frac{e}{2\pi}$. $\phi(L) = -\phi(R) = \frac{\theta_e}{2}$ is the curved angle for coordinates.

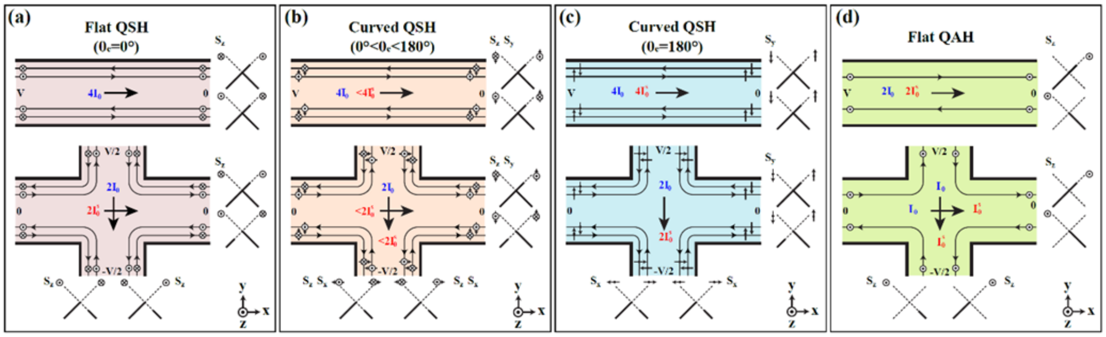

Supplementary Figure 4| Comparison of two-terminal and four-terminal measurement geometries for a (a) flat QSH system (θ_e =0°), (b) curved QSH system (0<θ_e<180°), (c) curved QSH system (θ_e =180°), and (d) QAH system. The arrows indicate the charge current I and spin current I^s and their flow directions. The unit of I and I^s are $I_0=(e^2/2h)V$ and $I_0^s=(e/8\pi)V$, respectively. The diagrams in the right and bottom indicate the population of the edge states. In terms of transport, with two terminals, the flat QSH device conducts only a charge current I with no spin current I^s, while the curved QSH and QAH devices similarly conduct both charge and spin currents. With four terminals, the flat QSH device conducts a longitudinal charge current (I_l) and a transverse pure spin current ($I^s{}_t$), while the curved QSH device with 0<θ_e<180° conducts both longitudinal charge I_l and spin $I^s{}_l$ (only S_x spin component) currents as well as a transverse pure spin current $I^s{}_t$ (only S_z spin component). Interestingly, $I^s{}_t$ ($I^s{}_l$) continues to decrease (increase) with increasing θ_e, and $I^s{}_t$ vanishes at θ_e =180°. Similar to curved QSH device, four-terminal QAH device conducts both longitudinal charge and spin currents; but different from QSH device, QAH device conducts both transverse spin and charge currents. In terms of robustness against elastic back-scattering, the curved QSH is the same as the flat one protected by time reversal symmetry, which would be broken only if magnetic impurity were introduced. In terms of conductance quantization, in all the devices, charge currents are quantized in unit of $I_0=(e^2/2h)V$. However, spin currents are only quantized in unit of $I_0^s=(e/8\pi)V$ in the flat QSH and QAH devices but not in the curved QSH device, because in the latter the spin current is not conserved with varying longitudinal and transverse components and it can reach the maximum "quantized" value of $4I_0^s$ ($2I_0^s$) in two- (four-) terminal setting.

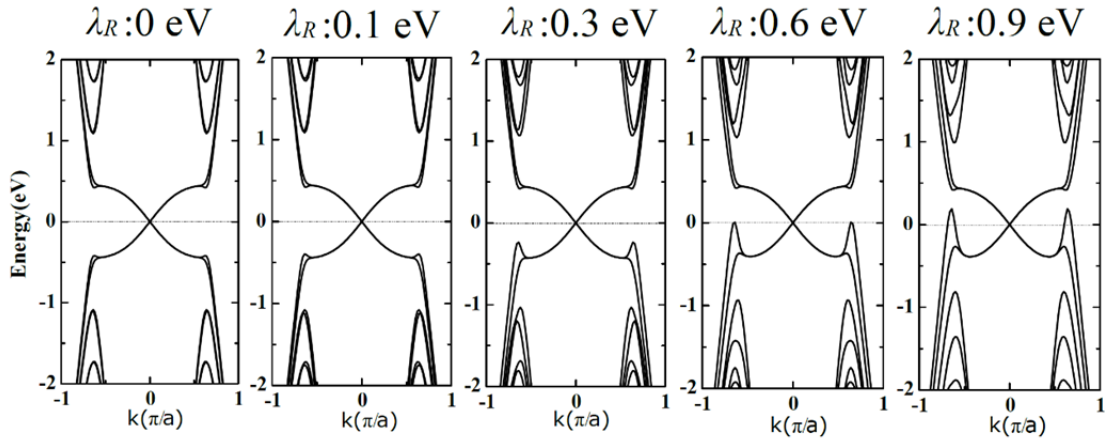

Supplementary Figure 5| The calculated electronic band structures of zigzag-edge QSH nanoribbons (θ_e=60°) as a function of λ_R. When λ_R is increased from 0 to 0.9 eV, the Rashba effect can significantly reduce the original SOC band gap, but the topological properties of the whole system are unchanged as long as the SOC band gap is not closed. Importantly, these calculations show that the Rashba effect will not change the spin orientation of edge states.

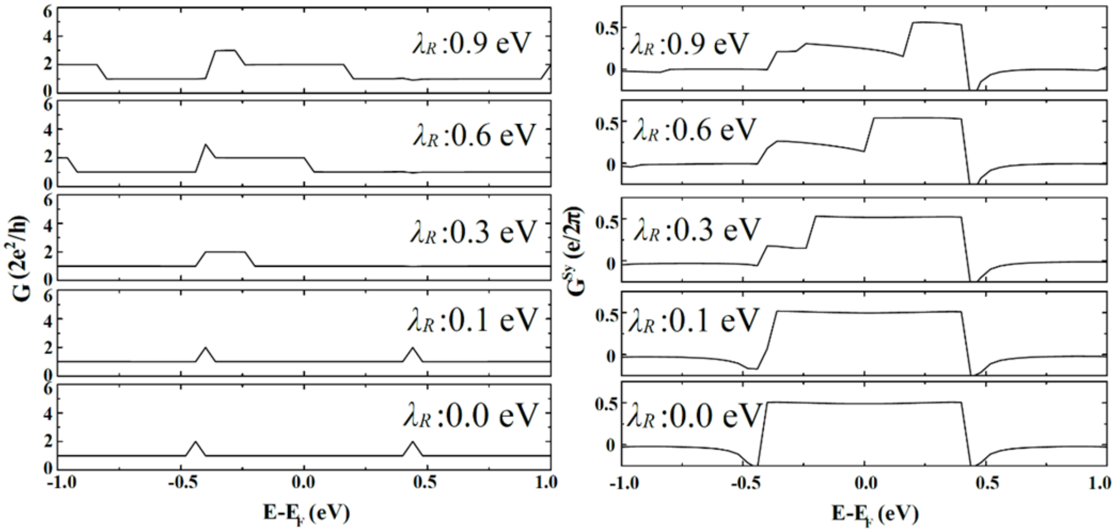

Supplementary Figure 6| The calculated charge (G) and spin (G^{sy}) conductance of the zigzag-edge QSH nanoribbons (θ_e=60°) as a function of λ_R. When λ_R is increased, the valence bands of the QSH nanoribbons split and shift upward. when $\lambda_R > 0.6$ eV, the Rashba effect can even push the bulk valence band merging into the Fermi level, which will in turn influence the plateau of charge conductance G and spin conductance G^{sy} around the Fermi level. However, it will not affect our main conclusion, i.e., the curvature can be applied to generate a non-zero spin conductance in a QSH system under a two-terminal device setting. It is should be noted that in real materials the Rashba effect

is usually much smaller than 0.6 eV. Thus, our main conclusion should not be affected significantly by the Rashba effect.

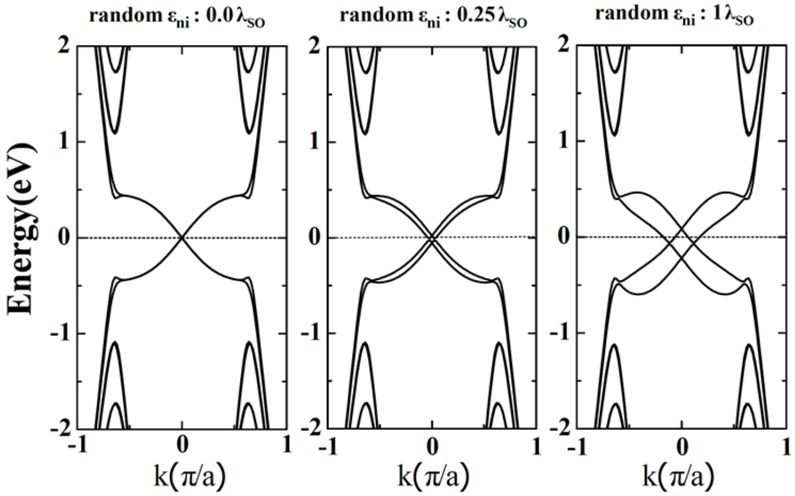

Supplementary Figure 7│ The calculated band structures of zigzag-edge QSH nanoribbons (θ_e=60°) as a function of different random ε_{ni}. We considered two cases by varying the on-site energies from the initial values up to 0.25 λso or 1.0 λso. Here we have randomly selected two configurations to calculate their electronic properties. As an example, this figure shows the calculated band structures for a bent QSH ribbon with bending angle θ_e =60°. The random on-site energies will effectively lift the degeneracy of edge states, but it will not affect the spin rotations of edge states.

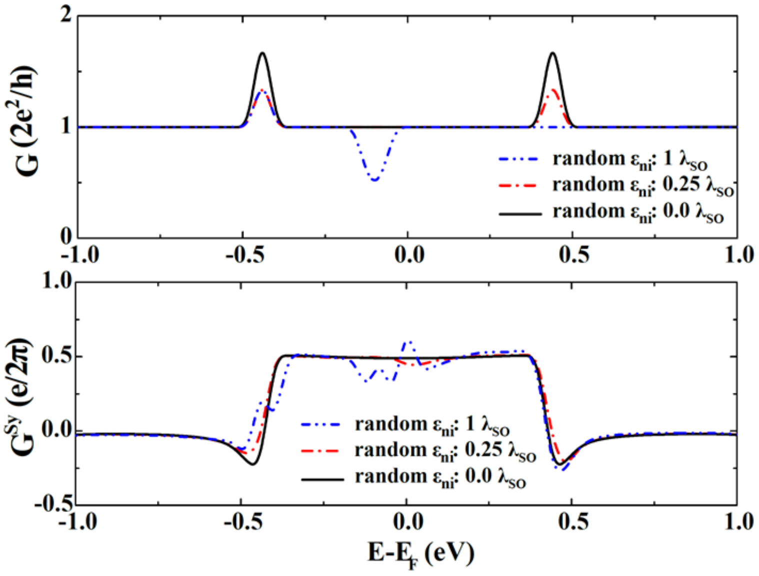

Supplementary Figure 8│ The calculated charge (G) and spin (G^{sy}) conductances of zigzag-edge QSH nanoribbons (θ_e=60°) as a function of different random ε_{ni}. The random on-site energies will make some quantitative change in the charge conductance G and spin conductance G^{sy} curves, but they will not change the overall shape of

conductance spectrums or alter our main conclusions.

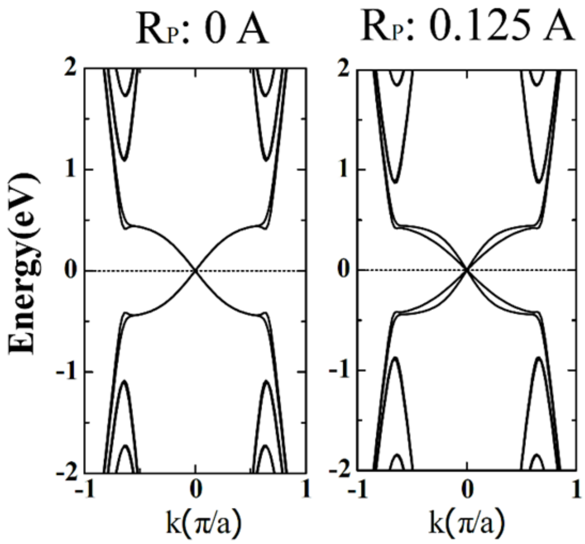

Supplementary Figure 9| The calculated electronic band structures of zigzzag-edge QSH nanoribbons (θ_e=60°) without and with random atomic position effect. We assume all the atoms are displaced from their equilibrium positions in any given direction by a maximum distance of 0.125 Å. This will effectively change the hopping term, $t_{mj,ni}$, even if the changes in (ppπ) and (ppσ) are negligible. Similar to the random-onsite-energy effect, the random-position-effect also lifts the degeneracy of edge states.

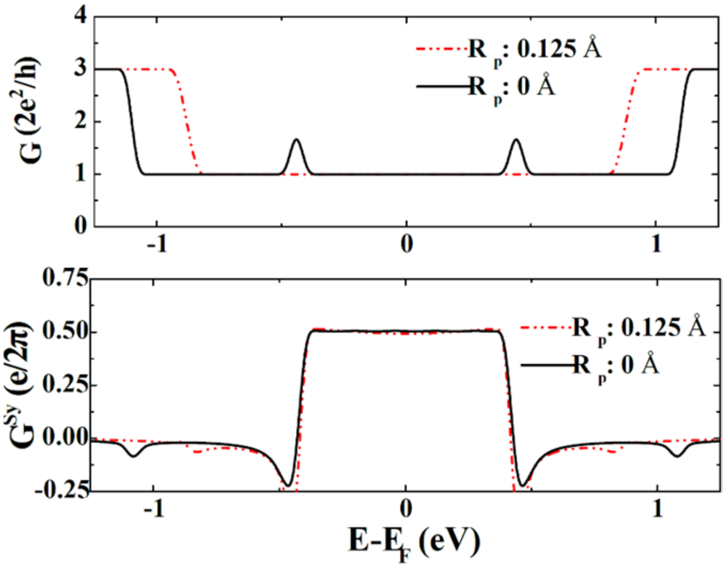

Supplementary Figure 10| The calculated charge (G) and spin (G^{sy}) conductances of QSH nanoribbons (θ_e=60°) without and with random atomic position effect. We have

randomly selected one configurations to calculate its electronic and transport properties. However, it will not affect the transport properties of QSH nanoribbons.

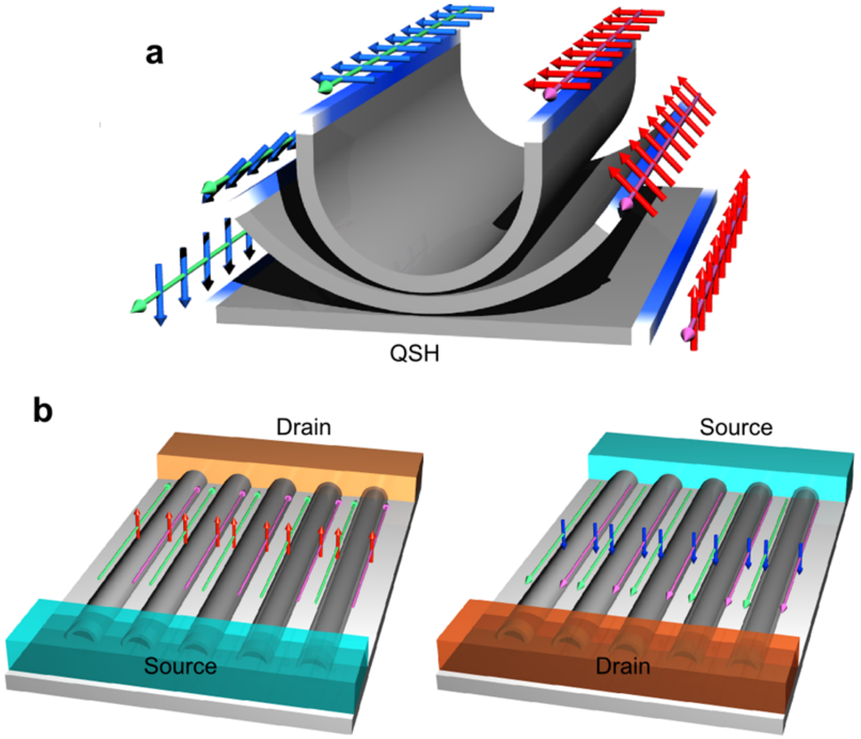

Supplementary Figure 11| Device concept of a spin injector produced by nanomechanical architecture process. (a) Schematics of a QSH state and their edge spin orientations at different bending curvatures. (b) Schematic design of a proposed spin injector device: mass production of self-rolled up QSH nanofilms on a substrate produced by the concept of nanomechanical architecture process, connected with electrodes. It is a parallel process that can facilitate mass production of identical partial cylindrical QSH arrays, which can function ideally as a robust spin injector device with high spin current density, while spin polarization can be switched by changing bias direction. Compared to the traditional magnetic materials, the QSH based spin injectors are topologically protected, robust against structural distortion or impurity scattering; the helical Dirac edge states support also ultrafast spin current.
